# Molecular architecture of mammalian pyruvate dehydrogenase complex

**DOI:** 10.1093/procel/pwae044

**Published:** 2024-08-24

**Authors:** Maofei Chen, Yutong Song, Sensen Zhang, Yitang Zhang, Xudong Chen, Minghui Zhang, Meng Han, Xin Gao, Sai Li, Maojun Yang

**Affiliations:** Tsinghua-Peking Center for Life Sciences, Beijing Frontier Research Center for Biological Structure, School of Life Sciences, Tsinghua University, Beijing 100084, China; Ministry of Education Key Laboratory of Protein Science, Tsinghua University, Beijing 100084, China; Tsinghua-Peking Center for Life Sciences, Beijing Frontier Research Center for Biological Structure, School of Life Sciences, Tsinghua University, Beijing 100084, China; State Key Laboratory of Membrane Biology, Tsinghua University, Beijing 100084, China; Tsinghua-Peking Center for Life Sciences, Beijing Frontier Research Center for Biological Structure, School of Life Sciences, Tsinghua University, Beijing 100084, China; Ministry of Education Key Laboratory of Protein Science, Tsinghua University, Beijing 100084, China; Tsinghua-Peking Center for Life Sciences, Beijing Frontier Research Center for Biological Structure, School of Life Sciences, Tsinghua University, Beijing 100084, China; Ministry of Education Key Laboratory of Protein Science, Tsinghua University, Beijing 100084, China; Tsinghua-Peking Center for Life Sciences, Beijing Frontier Research Center for Biological Structure, School of Life Sciences, Tsinghua University, Beijing 100084, China; Ministry of Education Key Laboratory of Protein Science, Tsinghua University, Beijing 100084, China; Department of Biochemistry and Molecular Biology, School of Basic Medical Sciences, Shenzhen University Health Science Center, Shenzhen 518060, China; Protein Research Technology Center, Protein Chemistry and Omics Platform, School of Life Sciences, Tsinghua University, Beijing 100084, China; Computer Science Program, Computer, Electrical and Mathematical Sciences and Engineering Division, King Abdullah University of Science and Technology (KAUST), Thuwal 23955-6900, Kingdom of Saudi Arabia; Computational Bioscience Research Center, King Abdullah University of Science and Technology, Thuwal 23955-6900, Kingdom of Saudi Arabia; Tsinghua-Peking Center for Life Sciences, Beijing Frontier Research Center for Biological Structure, School of Life Sciences, Tsinghua University, Beijing 100084, China; State Key Laboratory of Membrane Biology, Tsinghua University, Beijing 100084, China; Tsinghua-Peking Center for Life Sciences, Beijing Frontier Research Center for Biological Structure, School of Life Sciences, Tsinghua University, Beijing 100084, China; Ministry of Education Key Laboratory of Protein Science, Tsinghua University, Beijing 100084, China; Cryo-EM Facility Center, Southern University of Science and Technology, Shenzhen 518055, China; Beijing Life Science Academy, Beijing 102209, China

**Dear Editor,**

Pyruvate dehydrogenase complex (PDHc) is a large multienzyme assembly (Mr = 4–10 million Daltons) consisting of three essential components: pyruvate dehydrogenase (E1p), dihydrolipoyl transacetylase (E2p), and dihydrolipoyl dehydrogenase (E3). These three enzymes perform distinct functions sequentially to catalyze the oxidative decarboxylation of pyruvate with formation of nicotinamide adenine dinucleotide (NADH) and acetyl-coenzyme A (Patel and Roche, 1990). By irreversibly converting the main product of glycolysis to precursors of tricarboxylic acid cycle, PDHc controls the carbon flux in carbohydrate catabolism and act as a gatekeeper to maintain glucose homeostasis (Stacpoole and McCall, 2023). Abnormal PDHc activity is linked to severe metabolic diseases, including lactic acidosis, hypotonia, brain malformation, and neurodevelopmental delay (Ebertowska et al., 2020).

In eukaryotic cells, PDHcs predominantly reside in mitochondrial matrix, with an additional component known as E3-binding protein (E3BP, also termed as Protein X) (Neagle et al., 1989), which shares a remarkable similarity with E2p component in domain structure (Fig. 1A). Mammalian E2p and E3BP jointly assemble into a 60-mer icosahedral core scaffold with their C-terminal inner core (IC) domains (Patel et al., 2014). Exterior to the core, E1p and E3 are noncovalently anchored to the peripheral subunit binding domains (PSBD) of E2p and E3BP, respectively, forming a flexible outer shell around inner scaffold (Kyrilis et al., 2021). Between inside and outside, mobile lipoyl domains (LD) located at the N-terminal regions of E2p/E3BP shuttle around the active sites of E1p and E3, facilitating the transfer of various intermediates and enabling the whole enzymatic operation (Perham and Richard, 2000). Although individual subunits have been well characterized, the quaternary structure of intact PDHc remains a puzzle due to its inherent complexity. This limits our understanding about the spatial organization and dynamic catalysis mechanism of the system.

**Figure 1.**
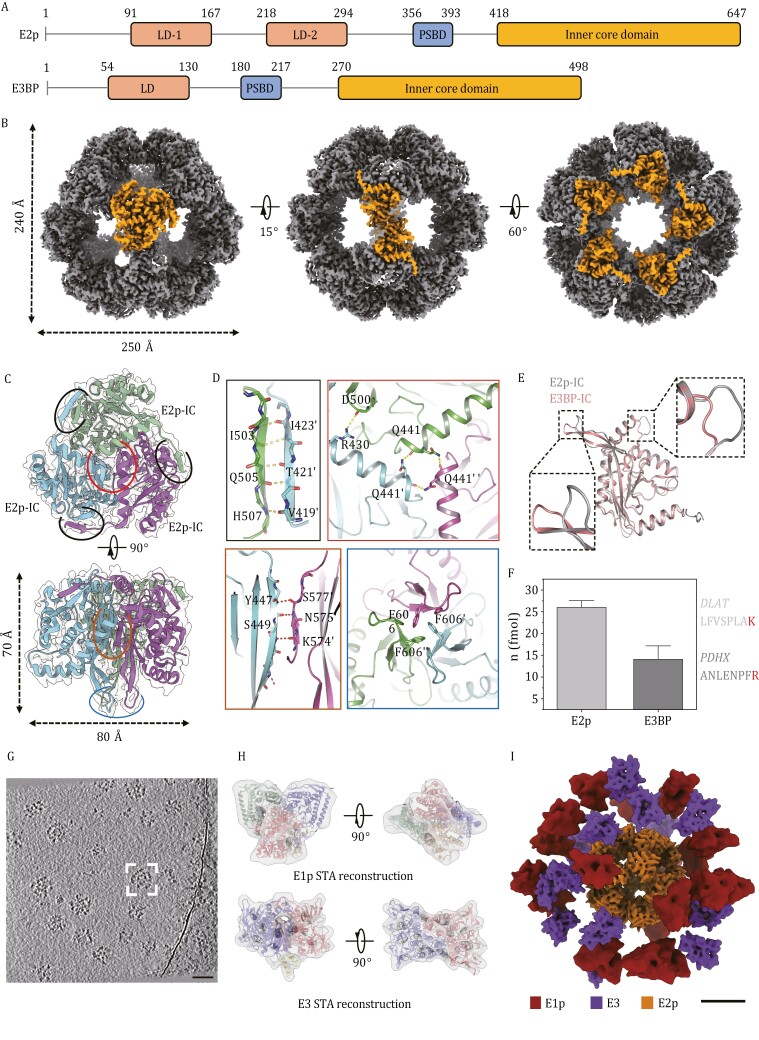
Structural characterization of *Sus scrofa* PDHc core and overall landscape of integrated complex. (A) Schematic representation of *Sus scrofa* E2p and E3BP organization, wherein each structural domain is indicated. Linker regions connecting these domains are displayed as lines. (B) Cryo-EM SPA reconstruction of the dodecahedral PDHc core scaffold across three views. The surface view along 3-, 2-, and 5-fold axes is illustrated from left to right with specific E2p monomers highlighted. (C) Structural model of E2p-IC homotrimer constructed from density map of scaffold vertex. Interaction sites within a single trimer are circled. (D) Interactions within E2p trimer. Four types of interfaces are marked with detailed binding sites magnified. (E) Structural comparison between the IC domain of porcine E2p and E3BP. The regions with relatively large variances, mainly in the loop area, are highlighted using dashed boxes in the superimposition. (F) Bar graphs of relative E2p/E3BP content in the hybrid PDHc core. Unique peptides of E2p (*DLST*) and E3BP (*PDHX*) are indicated with Isotope-labeled residues highlighted. Data are represented as means ± SEM using *n* = 3 independent mass spectral experiments. (G) A representative tomogram slice (5 Å thickness) exhibiting pleomorphic PDHc particles. Scale bar represents 50 nm. (H) Cryo-ET reconstructions of E1p, and E3 yield by subtomogram averaging. Density maps (35% surface transparency) are fitted with crystal structures of human E1p heterotetramer (PDB: 3EXE) and human E3 dimer (PDB: 2F5Z), respectively. (I) A representative complex (boxed in 1G) reconstructed by projecting E1p, E2p, and E3 onto refined coordinates. Scale bar represents 10 nm.

To investigate the overall architecture of native mammal PDHc, we have extracted endogenous PDH complexes from porcine (*Sus scrofa*) myocardium based on a conventional method of mitochondrial multienzyme isolation (Fig. S1A). For further purification, an optimized density gradient centrifugation was then performed on the crude extract (Fig. S1B), from which the PDHc fractions containing all essential components were identified and collected (Fig. S1C). Microscopy observations revealed that purified protein complexes exhibited a nearly spherical shape with a particle diameter ranging from 48 to 55 nm (Fig. S1D), consistent with the morphological features of previously reported eukaryotic PDHcs (Gu et al., 2003). Activity assays, which target the end-product (NADH) accumulation, also confirmed that PDHc fractions possessed a complete catalytic capability of pyruvate oxidative decarboxylation (Fig. S1E and S1F), suggesting our purified product remains in its natural active state.

We then collected 11,575 cryo-EM micrographs of purified PDHc and processed the dataset with single-particle analysis (SPA). Averaging images from 2D classification revealed that while the internal densities maintained a regularly ordered configuration, the external densities appeared irregular and diffuse (Fig. S2A), indicating that peripheral subunits exhibited significantly mobility and were non-identical among complexes. We thereby masked the external densities and conventionally enforced ortho-icosahedral symmetry to determine the IC structure at a resolution of 3.66 Å (Fig. S2B–D; Table S1). Similar to those of *Homo sapiens* and *Bos taurus* (Liu et al., 2022), the porcine PDHc core presented a tightly stacked dodecahedral scaffold (Fig. 1B), consisting of 60 repeating IC domains, in which C-terminal region of E2p (residues 418–646) was appropriately fitted, whereas the N-terminal regions, including LD domains, PSBD domain, and their inter-domain linkers, were excluded due to their conformational flexibility (Fig. S3).

Within this reconstruction, adjacent IC domains are compactly trimerized to form a dodecahedron vertex, which visually resembled a tripetalous, pyramid-like structure with a height of 70 Å and a diameter of 80 Å (Fig. 1C), reminiscent of homologous structures in the cubic cores of *A. vinelandii* E2p (Hendle et al., 1995). Comparable intra-interactions were detected in porcine E2p vertex, wherein each trimer, with a total buried area of 4,272 Å^2^, incorporated multiple inter-domain interactions at four different regions to maintain structural stability (Fig. 1D). These twenty vertexes were organized into scaffold assemblies through hydrophobic interactions, with a classical double-handed manner. This characteristic “knob-hole” combination linked neighboring vertexes along 2-folded axes (Fig. S4), forming the dodecahedral edges with a side length ~80 Å.

Unlike those in prokaryotes or fungi, the constituents of mammalian PDHc cores encompass not only E2p but also an indeterminate number of E3BP. Nonetheless, the sequence homology between porcine E2p and E3BP at the C-terminal fragment (Fig. S5) results in nearly identical IC domain structures (only slight disparities in loop regions) (Fig. 1E), rendered it challenging to place E3BP in the final 3D map. To establish an accurate stoichiometry ratio between mammalian E2p and E3BP, we synthesized two unique peptides from porcine E2p (*LFVSPLAK*) and E3BP (*ANLENPFR*), respectively and labeled them with heavy atoms (^14^N and ^13^C) to serve as internal references for isotopic mass spectrometry. The specific value of E2p:E3BP determined by quantitative MS analysis approached approximately 2:1, with a range from 1.9:1 to 2.4:1 (Fig. 1F). These results indicated a “40 E2p + 20 E3BP” composition pattern, deviating from the stoichiometry of the hypothetical 48:12 model but providing substantial support for the 40:20 model (Brautigam et al., 2009).

Given the non-uniformity of peripheral subunits distribution, we utilized cryo-electron tomography (cryo-ET) to elucidate the integrated structure of *Sus scrofa* PDHc (Fig. 1G). This process was initiated by collecting tilt series of both purified PDHc protein samples and *in-situ* PDHc inside porcine mitochondria. The consistency between *in-situ* PDHcs and purified complexes, shown in reconstructed tomograms, confirmed that cryo-ET sample preparation of purified PDHc does not affect the integrity of complex (Fig. S6). Considering the low mitochondrial PDHc concentration, we chose to use purified PDHc for subsequent data processing. A total of 496 core subunits and 15,650 peripheral subunits were then manually selected and subjected to subtomogram averaging (STA) reconstruction (Fig. S7; Table S2). In particular, the core subunits, marked by distinctive pentagonal features, were grouped together and averaged with icosahedral symmetry, yielding a 11.7-Å resolution map that closely resembled the rigidly fitted SPA structure of E2p-IC scaffold (PDB ID: 7UOM), except for an uncovered N-terminal fragment (residues 420–426) (Fig. S8). Peripheral subunits, referring to E1p and E3, were classified into two corresponding clusters, and averaged separately to obtain a 13.4 Å cone-shaped E1p structure and a 10.2 Å bone-shaped E3 structure. We likewise fitted homologous crystal structures (PDB ID: 3EXE, 2F5Z) into the cryo-ET maps of E1p and E3 as a rigid body (Fig. 1H), with a resulting cross-correlation coefficient of 0.92 and 0.87, respectively, indicating our STA reconstructions faithfully represented the essential structural characteristics of mammalian PDHc components.

After back-projecting the component reconstructions into the original tomograms (Method details), we modeled a set of integrated complexes (*n* = 389) to directly visualize the authentic porcine PDHcs. Inspection of each individual complex outlined an amorphous architecture. As a whole, each core scaffold was observed to be unequally decorated with an average of 34 ± 9 peripheral subunits, akin to a model resembling a planet orbited by satellites (Fig. 1I; Video S1). Linkers connecting peripheral and core subunits, due to their inherent low signal-to-noise ratio, remained invisible, and creating an annular gap between inner and outer components. Within the external area, E1p and E3 appeared scattered and disorganized, with no definite interaction or combination modes between peripheral subunits identified.

A comprehensive data analysis of detailed structural information has been conducted for interpretation of such diversity. Firstly, E1p and E3 were counted separately in each PDHc entity (Fig. S9). The copy number of E1p per complex mainly fell within the range of 17–24. On the other hand, the majority of E3 copy numbers ranged from 8 to 16 (Fig. 2A). This wide range of quantitative changes undeniably contributes to the structural heterogeneity. In addition, each complex contained an average of 20.8 copies of E1p and 13.3 copies of E3, resulting in a quantitative ratio of E1p to E3 of approximately 1.6:1. This statistical ratio (E1p:E3 = 1.6:1) deviates from the theoretical ratio (E2p:E3BP = 2:1) measured by our rescaled isotope mass spectrometry, suggesting there might be additional connectivity mechanisms besides the classic one-to-one PSBD combinations (Frank et al., 2005). Secondly, we calculated the occupancy of core subunits to assess the saturation of our complexes (Fig. 2B). Statistical results across all samples indicated that the proportion of combined core subunits in each complex was estimated to be approximately 57%. This level of saturation is comparable to that of wild type (WT) bovine heart PDHc (~50%), but about 1.5 times more than WT bovine kidney (~38%) (Zhou et al., 2001) and 1.3 times more than *C. thermophilum* (~44%) (Kyrilis et al., 2021). Thirdly, we measured the particle sizes of PDHcs as irregular geometric forms with point-selecting method. Distances from the geometric center of each E1p/E3 to that of core frame, representing the radius of different radial directions, were recorded and plotted (Fig. 2C). Surprisingly, we discovered that the interval spacing of E1p-to-core ~*N* (μ = 21.04, σ^2^ = 17.98) and E3-to-core ~*N* (μ = 21.11, σ^2^ = 20.61) were essentially consistent and both followed the Gaussian distribution, primarily ranging from 8 to 33 nm. This considerable range in dimensions span would significantly enhance the anisotropism of PDHc construction.

**Figure 2.**
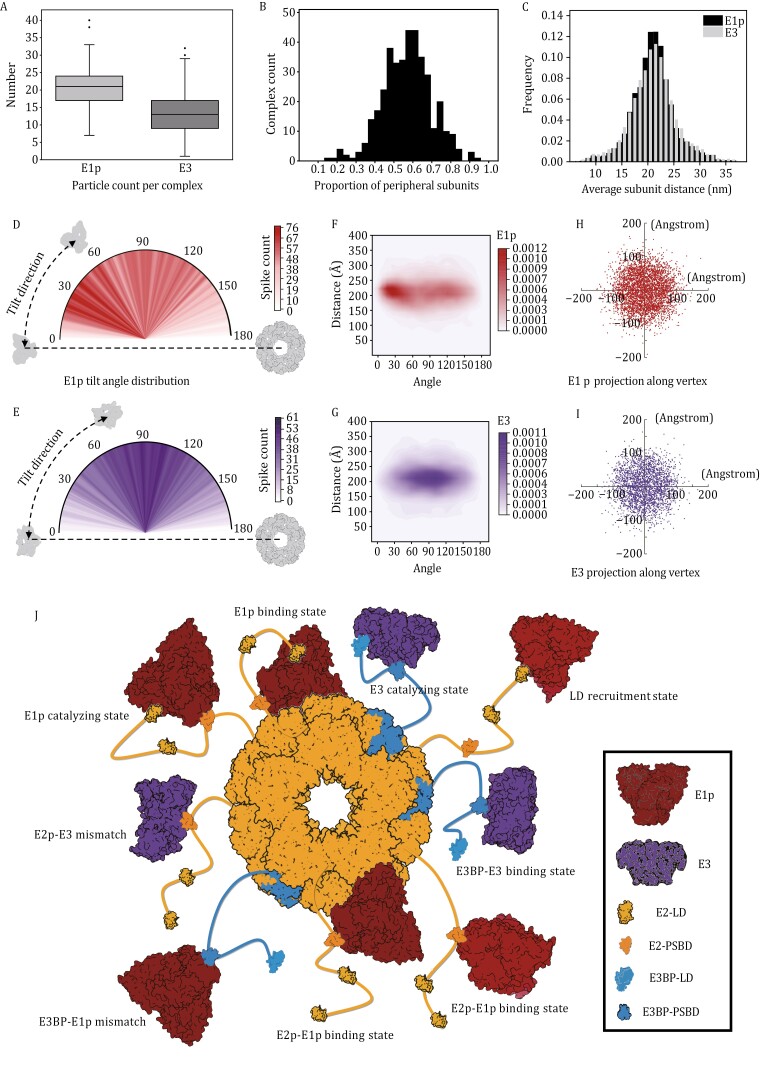
Spatial organization of peripheral subunits, with a proposed assembly mechanism of mammalian PDHc. (A) Number of E1ps and E3s identified per complex. The maximum, minimum, first quartile, medium, third quartile, and outliers of the data are indicated in boxplots. (B) Occupancy of the core subunits. The majority of the core scaffold in reconstructed particles is decorated by 40%–80% peripheral subunits, and the average core occupancy is estimated to be 57%. (C) Distance between peripheral subunits and core center. Distance^E1p-to-core^ and distance^E3-to-core^ share identical distribution patterns, and both fit the normal distribution properties with a mean value of 220 Å. (D and E) Polar plots illustrating the broad orientation distribution of E1p (2D) and E3 (2E) concerning the core. The semicircle represents the range of angular variation (step size: 1°) and chromatic value represents peripheral subunit count level. An example showcasing a 0° orientation and subunit tilt direction is provided using corresponding structural models. (F and G) 2D kernel density plots of distance and orientation data, showing tendentious distribution of E1p (2F) and E3 (2G). The darker shaded regions correspond to areas with a higher frequency of peripheral subunits. (H and I) Scatter plots on location statistics, indicating a uniform distribution of E1p (2H) and E3 (2I) in relation to the nearest vertex. Each data point in both plots represents a projection of the center-of-mass of E1p or E3 along the *Z* axis onto the *x*–*y* plane. (J) The integrated assembly model of mammalian PDHc unveils a flexible architecture. The E1p and E3 are positioned in the outer shell surrounding a hybrid core consisting of E2p and E3BP. E3BPs are randomly positioned within the E2p core due to the absence of definitive structural evidence. All units depicted in the model are based on high-resolution structures, such as porcine E2p core structure, human E1p (PDB: 3EXE), human E3 (PDB: 2F5Z), E2p-LD (PDB: 1IYU), E2p-PSBD (PDB: 1W88), E3BP-LD (AlphaFold2-Complex prediction), and E3BP-PSBD (PDB: 2F60). The structures of the flexible linker regions connecting E2p/E3BP domains are sketched from predicted models for illustrative purposes.

We next introduced a quasi-polar coordinate system to characterize the concrete spatial arrangement of peripheral subunits in global architecture (Fig. S10, Method details). As for angular distributions, refined orientations of E1p components showed that they rotate about their respective inner core almost randomly (ranging from 0.4° to 178.8°) without any specific patterns, except for a somewhat inconspicuous preponderant sector centered around 30° ± 15° compared to the rest (Fig. 2D). A similar distribution is observed in the angular statistics of E3 (ranging from 0.25° to 178.07°), although E3 exhibits a more concentrated range of rotation, leaning towards an average angle of 95° ± 25° (Fig. 2E). Such widespread distributions, also found in early cryo-ET studies of the *B. stearothermophilus* PDHc (Lengyel et al., 2008), were facilitated by the intrinsic mobility of the hinge linker fragments, which cannot be completely folded into a stable structure but exist in the form of flexible loops.

The comprehensive distributions, combining orientation and distance information (Fig. 2), were then rendered in a kernel density map to systematically reveal their distributed trends. Despite in highly dynamic status, a significant number of E1p and E3 were restricted to certain confines. We observed that E1p gathered in two primary spatial regions: (a) nearly 50% of E1p subunits were concentrated within a 19–24 nm interval from the complex center, with a tilt angle ranging from 15° to 45°; (b) around 25% of E1p dispersed in the range of 20–23 nm and rotated within the range of 75°–150° (Fig. 2F). In contrast, the arrangement of E3 subunits was more concentrated, primarily occupying a single dominant region (18–22 nm, 70°–125°) (Fig. 2G). Almost 60% of E3 was normally distributed within this main interval, with the remaining portion evenly radiating outward. The precise mechanism underlying these distribution tendencies is yet to be fully understood, but it may contribute to narrow down LD’s trail on a degree and partly increase the catalytic efficiency of integrated PDHc reaction, according to the substrate channeling model.

To further investigate in local, the 20 vertexes of each dodecahedral core were superimposed, and each neighboring peripheral subunit was adjusted to maintain its relationship to the vertex. We then examined these apical areas using a new coordinate system (Fig. S10). Both E1p and E3 were observed to be uniformly scattered around the vertex along the *x*–*y* plane (Fig. 2H and 2I). No fixed positional relationship was found around the corner, thus providing no clear evidence to support the hypothesis that E3 is located above the opening of the core. The tether lengths between vertex and peripheral subunits, which represent their connection distance, should fall within a certain range based on the typical PSBD binding modes. If the tether peptides are fully extended (0.38 nm/residue), for instance, the maximum allowable span length for E1p and E3 would be 9.12 nm (24 amino acids) and 19.76 nm (52 amino acids), respectively. Taking into account the ~3.5 nm radius, the center-of-mass of E1p was supposed to locate within 13 nm away from the tip of E2p-IC, and the maximum distance of E3 should not exceed 23 nm. A noteworthy finding in our reconstructions is that while most E3 projections remain within the tether threshold, nearly 20% of E1p projections exceed this limit (Fig. S11). In light of the discrepancy between E1p:E3 and E2p:E3BP ratios mentioned earlier, this overstepping scenario may be attributed to the presence of additional E1p binding sites located further towards the N-terminal region than the PSBDs in E2p sequence.

To fully explore how peripheral subunits bind to the core scaffold, we detected the interactions between E1p/E3 and the putative binding domains of E2p/E3BP (E2p-LDs, E2p-di, E2p-PSBD, E3BP-LD, and E3BP-PSBD) separately (Fig. S12A). The results indicate that both E2p-LDs have the ability to combine with E1p, although their binding affinity is noticeably weaker compared to that of E2p-PSBD. This suggests that E2p-LD could potentially serve as a possible candidate for extra E1p binding sites. Furthermore, we found that E2p-PSBD could slightly combine with E3, while E3BP-PSBD is also capable of binding E1p conversely. This seemingly mismatched situation might arise from the homologous structure of both PSBDs, which share conserved residues (Pro^133^ and Pro^154^ in human) that form a hydrophobic patch to participate in binding with the peripheral subunits (Ciszak et al., 2006).

Combining the known PSBD binding modes with our detection results, we propose a comprehensive assembly mechanism for PDHc components, which comprises six combinations between internal and external components (Fig. S12B): (i) E1p-E2p_PSBD binding state (the primary binding form of E1p in the complex, with a significant number of E1p subunits connecting to the core frame this way); (ii) E3-E3BP_PSBD binding state (the primary binding form of E3 in the complex, with a substantial number of E3 subunits connected to the core frame in this manner); (iii) E3-E2p_PSBD mismatch state (a small fraction of E3 is connected to the core via E2p_PSBD); (iv) E1p-E3BP_PSBD mismatch state (a small fraction of E1p is connected to the core through E3BP_PSBD); (v) E1p-E2p_LD recruitment state (a small amount of free E1p initially forms weak interactions with E2p_LD located at the outermost layer of the core scaffold); and (vi) E1p-E2p intermediate state (with the swinging motion of E2p_Linker, E1p attached to E2p-LD comes into close proximity with PSBD, forming stable binding). In conclusion, this model establishes a holistic framework for mammalian PDHc (Fig. 2J), highlighting its diverse assembly patterns that not only further increase the heterogeneity of the intact PDHc complex, but also enhance the adaptability and efficiency of this catalytic machine.

## Supplementary data

Supplementary data is available at *Protein & Cell* online at https://doi.org/10.1093/procel/pwae044.

pwae044_suppl_Supplementary_Materials

pwae044_suppl_Supplementary_Video_S1

## Footnotes

We thank the Tsinghua University Branch of China National Center for Protein Sciences (Beijing) for providing the cryo-EM facility support. We thank Dr. XiaoMin Li and Dr. Fan Yang for data collection; the computation was completed on the Yanglab GPU workstation.

M.Y. and S.L. directed the study. M.C. and M.Z. did the protein purification. M.C., M.Z., and Y.Z. performed cryo-EM sample preparation, data collection, and structural determination with help of S.Z.; M.H. performed the isotopic mass spectrometry. M.C. performed the enzymatic activity assay with the help X.C.; Y.S. and S.L. performed cryo-ET sample preparation, data collection, and structural determination. M.Y., S.L., M.C., and Y.S. built the model and drew the figures. M.Y., S.L., M.C. Y.S., S.Z., Y.Z., and X.G. wrote the manuscript. All authors contributed to the discussion of the data and editing the manuscript.

The 3D cryo-electron microscopy density map of E2p has been deposited in the Electron Microscopy Data Bank (EMDB: 37969). The coordinates of atomic model of E2p have been deposited in the Protein Data Bank (PDB: 8X03). Cryo-ET subtomogram averaging maps were deposited in Electron Microscopy Data Bank under accession codes: E1p (EMDB: 38162), E2p (EMDB: 38163), and E3 (EMDB: 38164). The authors declare no competing interests and agree to publish and participate.

This work was supported by the National Key R&D Program of China (2022YFA1302701), the National Natural Science Foundation of China (32030056 to M.Y.; 32241031 and 32171195 to S.L.), the scientific project of Beijing Life Science Academy (2023300CA0090), Tsinghua University Initiative Scientific Research Program (2023Z11DSZ001), the King Abdullah University of Science and Technology (KAUST) Office of Sponsored Research (OSR) under Award (OSR-2020-CRG9-4352), and Office of Research Administration (ORA) under Award No. URF/1/4352-01-01, FCC/1/1976-44-01, FCC/1/1976-45-01, REI/1/5234-01-01, and REI/1/5414-01-01.
