# Supplementary material for: Molecular architecture of mammalian pyruvate dehydrogenase complex
Source: Protein Cell. 2024 Aug 24;16(1):72–8. doi: 10.1093/procel/pwae044 (PMC11700594; doi:10.1093/procel/pwae044)
Supplement: pwae044_suppl_Supplementary_Materials [file pwae044_suppl_supplementary_materials.pdf]

# Supplementary materials

## Molecular architecture of mammalian pyruvate dehydrogenase complex

Maofei Chen[1,2,*], Yutong Song[1,3,*], Sensen Zhang[1,2,*], Yitang Zhang[1,2,*], Xudong Chen[1,2], Minghui Zhang[4], Meng Han[5], Xin Gao[6,7], Sai Li[1,3,#], Maojun Yang[1,2,8,9,#]

[1]*Tsinghua-Peking Center for Life Sciences, Beijing Frontier Research Center for Biological Structure, School of Life Sciences, Tsinghua University, Beijing 100084, China*

[2]*Ministry of Education Key Laboratory of Protein Science, Tsinghua University, Beijing 100084, China*

[3]*State Key Laboratory of Membrane Biology, Tsinghua University, Beijing 100084, China*

[4]*Department of Biochemistry and Molecular Biology, School of Basic Medical Sciences, Shenzhen University Health Science Center, Shenzhen, China*

[5]*Protein Research Technology Center, Protein Chemistry and Omics Platform, School of Life Sciences, Tsinghua University, Beijing 100084, China*

[6]*Computer Science Program, Computer, Electrical and Mathematical Sciences and Engineering Division, King Abdullah University of Science and Technology (KAUST), Thuwal 23955-6900, Kingdom of Saudi Arabia*

[7]*Computational Bioscience Research Center, King Abdullah University of Science and Technology, Thuwal 23955-6900, Kingdom of Saudi Arabia China*

[8]*Cryo-EM Facility Center, Southern University of Science & Technology, Shenzhen, China.*

[9]*Beijing Life Science Academy, Beijing 102209, China*

*These authors contributed equally to this work.

#To whom correspondence should be addressed:

Maojun Yang: maojunyang@tsinghua.edu.cn

Sai Li: sai@tsinghua.edu.cn

This PDF file includes:

Materials and Methods

Figs S1-S12 and Figure legends

Tables S1 and S2

# Methods

**Isolation of mitochondria from porcine heart**

The following procedures were conducted on ice or at 4 °C. Fresh porcine heart tissue was obtained from the slaughterhouse and taken to the laboratory promptly. 150 g of heart muscles were isolated from the fascia and vessels, and subsequently sectioned into small cubes approximately 1 $cm^3$ in size. These muscle tissues were carefully washed two times with Milli-Q water to remove the blood and suspended in 200 mL of buffer A (100 mM Tris pH 7.4; 225 mM sorbitol; 60 mM potassium chloride; 1 mM EGTA; and 0.1% BSA). The tissue suspension was homogenized using a large-capacity electric blender for 300 seconds until muscles were thoroughly pulverized. The homogenate was centrifuged at 3,000 × *g* for 20 minutes to remove cellular debris in the pellet. The supernatant was decanted and applied to ultracentrifugation at 20,000 × *g* for 30 minutes to obtain the crude mitochondrial pellet, which was subsequently suspended in 100 mL of buffer B (50 mM Tris, pH 7.4; 250 mM sucrose; 60 mM potassium chloride; 40% Percoll; and 0.1 mM EGTA). This mixture was centrifuged at 60,000 × *g* for 50 minutes. The mitochondrial band was removed carefully and diluted with 20 mL of buffer C (50 mM Tris-base, pH 7.4; 100 mM sorbitol; 60 mM potassium chloride; and 0.05 mM EGTA). Ultimately, pure mitochondria were obtained following centrifugation of this mixture for 30 minutes at 20,000 × *g*.

**Purification of pyruvate dehydrogenase complex**

Purified mitochondria were suspended in 200 mL of buffer K (25mM potassium phosphate, pH 6.5; 10mM sodium chloride; 1%PMSF) and crushed using a high-pressure cell disrupter three times. The lysate was centrifuged at 60,000 × *g* for 30 minutes to eliminate the insoluble mitochondrial membrane. A dropwise addition of 15% (w/v) PEG-6000 to the supernatant was conducted, and the mixture was incubated for 10 minutes at room temperature for precipitation of OADH complexes. The crude extract as sediment was collected by ultracentrifugation at 150,000 × *g* for 30 minutes. This extract was then suspended in 50 mL of buffer D (50 mM MOPs, pH 7.2; 1 mM magnesium chloride; 20 mM DTT). After dissolution at 4 °C for 12 hours, the resulting supernatant was concentrated to a volume of 6mL using a 100-kDa cutoff centrifugal filter (Millipore). Then, every milliliter of concentrated sample was centrifuged on a 0.3 to 1.0 M sucrose cushions in 11.5 mL buffer S (25 mM Tris, pH 7.4; 20 mM potassium chloride) at 150,000 × *g* for 20 hours at 4 °C using an SW41 rotor (Beckman Coulter). Gradients were fractionated (1 mL per gradient) and sequentially assessed by 10% SDS-PAGE and MS analysis. Fractions containing PDHc components were identified and pooled. Following concentration to a volume of 1.5 mL, these PDHc fractions were subjected to final

purification via gel filtration chromatography (Superose 6, 10/300 GL, GE Healthcare) in buffer D. The peak fractions were compiled for activity detection and electron microscopy examination.

**Enzyme activity assays**

In order to assess the activity of purified porcine PDHc, a reaction mixture (0.2 mL) containing 50 mM MOPs (pH 7.0), 100 mM sodium chloride, 3 mM $NAD^+$, 0.4 mM Coenzyme A, 2 mM magnesium chloride, 2 mM DTT, 2 mM ThDP, and 20 µg purified PDHc sample was assembled. After activation of the mixture at 30 °C for 1 minute, the reaction was initiated by the addition of 1% (v/v) pyruvate stock solution. Enzyme activity was measured by continuously monitoring the increasing UV absorption of the overall reaction product, NADH (characteristic absorption peak at 340nm), in 96-well plates (PerkinElmer, USA) at 37 °C for 35 minutes. The spectrophotometric data were recorded by microplate reader (Envision-II, Revvity) at 60-second intervals. To obtain the *K*m value of wild type PDHc, increasing concentrations of substrate (0.05 mM- 2 mM) were added to the reaction mixture. Each group of experiments was repeated three times and the results were analyzed by GraphPad PRISM-10. The *K*m values of purified PDHc samples were determined using non-linear regression.

**Cryo-EM data acquisition**

Cryo-EM grids were prepared using an FEI Mark IV Vitrobot (Thermo Fisher Scientific). Aliquots with a volume of 4 µL of purified PDHc at a concentration of 1.5 mg/mL were added to glow-discharged 400-mesh Quantifoil R-1.2/1.3 grids (Quantifoil, Micro Tools, Germany). After 30 seconds, the grid was blotted (8 °C, 100% humidity, 1 second blotting time, and -1 blotting force) and plunge-frozen in liquid ethane. Grids were loaded into a Titan Krios 300 kV (Thermo Fisher Scientific) equipped with a Gatan Imaging Filter (GIF) Quantum LS and a Gatan K3 Summit direct electron detector. Movies were recorded using SerialEM in super-resolution mode. The nominal magnification was 130,000 ×, corresponding to a calibrated pixel size of 1.0975 Å at the specimen level. The total exposure time in each movie was established as 8 seconds, and fractionated equally into 48 frames, resulting in a total dosage of ~48 electrons/$Å^2$. Defocus was set from -1.5 to -2.5 µm.

**Cryo-EM data processing**

The movies were motion-corrected using UCSF MotionCor2 (Zheng et al., 2017). The contrast transfer function (CTF) parameter was determined using Gctf (Zhang, 2016) software based on the compiled micrographs. Following CTF estimation, we used a subset selection program in RELION-3.0 (Zivanov et al., 2018) in CTF selection mode to eliminate micrographs with a resolution lower than 4.5-Å and presenting with ice crystal diffraction rings. In this

way, 9,652 good-quality micrographs were selected from a total of 11,575 for further processing. Subsequent data analysis was conducted using the single-particle mode in RELION-3.0.

For intact PDHc complex, we set the box size to 720 pixels and a mask diameter of 65 nm to ensure external densities were entirely included. About 1200 particles with distinct PDHc characteristics were manually selected from 150 micrographs and subjected to 2D classification to obtain templets for particle auto-picking. In total, 126,283 particles were automatically selected by this templet. Following three rounds of 2D classification with a T value of 2 and classes of 100, 48,616 optimal particles with obvious densities of both peripheral and core subunits were identified. We then performed a single round of 3D classification with C1 symmetry using a sphere model as the initial reference and 3D auto-refine with the same symmetry using a 3D classification result model as the reference, resulting in a 32-Å resolution map according to a 0.143 cutoff criterion upon FSC calculation. To obtain high-resolution complex reconstruction, icosahedral symmetry was utilized as described, yielding an 18-Å resolution map in which peripheral subunits remain obscure.

For PDHc core reconstruction, the mask diameter was established as 280 nm to remove the interference of heterogeneous external densities. We used the same particle dataset from the previous auto-picking for four rounds of 2D classification and retained 73,566 best particles possessing prominent structural details of highly symmetrical core scaffolds. These particles were recentered and extracted over two rounds of 3D classification with conventional icosahedral symmetry (K=3, T=4). Classes containing regular dodecahedron geometry were sub-selected for auto-refinement with a solvent mask, resulting in a 4.58-Å resolution map. Following CTF refinement and postprocessing, we determined the PDHc core structure at 3.66-Å resolution. Detailed data processing steps and parameters are outlined in Extended Data Fig. 2.

**Model building and refinement**

The atomic model of PDHc core was built by cryo-Net (Xu et al., 2019) using neural network based method. Owing to the flexibility of the N-terminal linker, only residues in the ICD were built. The initial build model was firstly refined manually in coot-0.9.7 (Emsley and Cowtan, 2004) according to the map. Real-space refinement was performed in Phenix (Adams et al., 2010) to build the final atomic model. USCF chimera X and Pymol were used to analyze the map and atomic model, respectively. Refinement parameters was recorded in Table S1.

**Cryo-ET data acquisition of purified PDHc**

Cryo-ET grid preparation shared common equipment and operation methodologies with Cryo-EM, except that a 1 μL drop of gold fiducial beads (10 nm diameter; Aurion, the Netherlands) was mixed into 3 μL of PDHc protein sample (2.0 mg/mL) before loading into the Vitrobot. Cryo-ET data was recorded on a Titan Krios microscope (Thermo Fisher Scientific, Hillsboro, OR) operated at 300 kV equipped with an energy filter (slit width 20 eV; GIF Quantum LS, Gatan, CA) and K2 summit detector. Tilt series were collected in super-resolution mode at a nominal magnification of 105,000 ×, corresponding to a calibrated pixel size of 0.665 Å. Tilt series composed of 41 movies between -60º to 60º, 3º per step were acquired using bidirectional scheme by serialEM (Mastronarde, 2005). Each Movie with 8 frames in 0.1 s/frame exposure time and a dose of 3.194 e/Å$^2$ was recorded in a defocus range from -2 to -4 μm.

**Cryo-ET reconstruction of PDHc components**

Frames were motion-corrected and averaged using MotionCor2(Zheng et al., 2017). Defocus of movies were estimated by Gctf (Zhang, 2016). Movies at each tilt were filtered according to the cumulative dose (Grant and Grigorieff, 2015). Tilt series alignment was processed in IMOD by tracking fiducial markers. Stacks were contrast transfer function corrected in NovaCTF (Turoňová et al., 2017) and tomograms were reconstructed through weighted back-projection. 94 tomograms were binned by 4 × and low-pass filtered to 80 Å for better visualization. In total, 15,650 peripheral subunits and 496 inner cores were manually picked.

Dynamo (Castaño-Díez et al., 2012) was used for subtomogram averaging. For peripheral subunits, 15,650 manually identified particles in boxes of 48 × 48 × 48 voxels were extracted from 4 × binned tomograms. The initial template was generated by averaging the 15,650 subtomograms and low-pass filtered to 40 Å. Two iterations of alignment imposing C1 symmetry were performed to center particles. Then, using multireference alignment imposing C1 symmetry, 9,701 E1p and 5,949 E3 particles were classified. Low-pass filtered to 25 Å maps derived from the crystal structure of E1 (PDB: 3EXE) (Kato et al., 2008) and E3 (PDB: 2F5Z) (Brautigam et al., 2006) were served as classification templates. After removing duplicates according to distance, 8,686 E1p and 5,560 E3 were re-extracted in 96 × 96 × 96 voxels from 2 × binned tomograms and subtomogram averaged using a customized "gold-standard adaptive bandpass filter" method (Yao et al., 2020). A criterion of 0.143 for Fourier shell correlation was used to estimate the resolution. At this stage, the average of 8,686 E1p particles restricted to 16 Å served as a template, and C1 symmetry was imposed in E1p alignment. The crystal structure (PDB: 2F5Z) filtered to 15 Å served as a template, and C2 symmetry was imposed in E3 alignment. For the inner core, 131 manually picked particles were extracted in

a box size of 64 × 64 × 64 from 4 × binned tomograms and averaged to generate an initial template. The 131 cores were subtomogram averaged, imposing C1 symmetry, to generate a low-resolution template. Then, 496 manually picked inner cores of 64 × 64 × 64 voxels were extracted from 4 × binned tomograms and aligned to the low-resolution template, applying icosahedral symmetry. Subsequently, 494 subtomograms of 128 × 128 × 128 voxels were re-extracted from 2 × binned tomograms for gold-standard alignment and icosahedral symmetry was applied. The two half maps from the gold standard alignment were aligned and the resolution was estimated in Dynamo with masks, using a cutoff criterion of 0.143. E1p, E2p, and E3 were estimated with resolutions of 13.4 Å, 11.7Å and 10.2 Å, respectively. An empirical B-factor of -1200 was applied to sharpen the core structure.

**Assembly structure reconstruction**

E1p, E2p, and E3 were projected and merged together according to the aligned coordinates and orientations. One representative pyruvate dehydrogenase complex was selected for presentation using UCSF Chimera (Pettersen et al., 2004) and Chimera X (Goddard et al., 2018). For other map projections to coordinates (Fig. 2), the ‘dtplot’ function in Dynamo was employed.

**Quantification and statistical analysis**

For number statistics of porcine PDHcs (Fig. 2A and Fig. S9), all 389 complexes of the sample set were used. Every E1p and E3 component within each reconstructed complex were counted and recorded, respectively. The occupancy of core subunits (Fig. 2B) was calculated via dividing the sum of E1p and E3 copy numbers per complex by 60 (the sum of E2p and E3BP). Under this circumstance, we defined that E2p/E3BP are fully occupied (100% occupancy) if they are combined with 60 E1p/E3, and conversely, 0% occupancy implies the absence of peripheral subunits.

For spatial distribution statistics (Fig. 2C-2E), distances between the 8,130 refined E1p centers and 389 icosahedral core centers, belonging to the same PDHc, were measured. Similarly, distances between 5,188 E3 and 389 core were measured. By using the definition of polar coordinate system as reference, a coordinate system was established to exhibit the angular distribution, wherein the geometric center of core scaffold was defined as the origin, and geometric centers of E1p and E3 as the arbitrary points. Vectors pointing from the refined icosahedral core center to the centers of E1p and E3 were defined as vectors E2p-E1p and E2p-E3, respectively. Angle between vector E2-E1p and the two-fold axis of E1p were measured. Likewise, the angle between vector E2p-E3 and the twofold axis of E3 were measured.

For local distribution analysis (Fig. 2H-2I and Fig. S11), a total of 386 PDHc including 8,060 E1p and 5,145 E3 were used for statistics. A coordinate system was established according to related articles (Lengyel et al., 2008), wherein the center-of-mass of vertex (~115.2 Å away from the core scaffold center) was set as the origin point, the 3-fold axis of vertex as the z-axis, and the direction of the x-axis left arbitrary. In each PDHc, the nearest vertex to each E1p were selected, and the refined E1p coordinate were projected onto the vertex plane, which is normal to the threefold axis of the core. E3 coordinates were projected in the same manner as E1p. The height from E1p to the vertex plane, and the distance between E1p projection and the origin point, were measured. E3 also follow the same process.

**Cryo-ET data processing of porcine mitochondria**

For sample preparation, the extracted porcine mitochondria, obtained from the purification process mentioned above, were added to the glow-discharged grid (Quantifoil R-2/2, Au, 200 mesh) and plunged into liquid ethane at 22 °C and 100% humidity (waiting time: 60 seconds; blot time: 8 seconds). The grids were loaded into a 300-KV FEI Titan Krios microscope with an energy filter and Gatan K3 summit direct-detection camera. Cryo-ET data of mitochondria was recorded in super-resolution mode at the nominal magnification (8,100 ×) with a calibrated pixel size of 0.75 Å. The tilt series was collected from 0° using a dose-symmetry scheme with a 3° angular increment between -60° and 60° by SerialEM. At each angle, an 8-frame movie was recorded with a dose of 123 e/Å$^2$ per tilt series. The defocus range was from -2 to -4.5 μm.

All of the micrographs were motion-corrected and CTF-estimated in Warp (Tegunov and Cramer, 2019). The tilt series were aligned by AreTomo (Zheng et al., 2022) and CTF-corrected by NovaCTF. Tomograms were generated by IMOD and were submitted to IsoNet (Liu et al., 2022) for compensating missing wedge. In the refined tomograms, the mitochondrial cristae, ATP synthase and a total of 35 in-situ PDHcs were identified based on their distinctive morphological characteristics. Peripheral subunits in each complex were manually selected and counted using dynamo. The number and proportion statistics of peripheral subunits were conducted as mentioned hereinbefore.

**Isotopic mass spectrometric analysis**

The isotopic mass spectrometric analysis of the hybrid E2p/E3BP core referred to quantitative proteomic analysis of E2p-E3BP by LS-MS. E2p and E3BP components were separated via SDS-PAGE followed by in-gel digestion for subsequent MS investigation. Specifically, all proteins were treated with 25mM dithiothreitol (DTT) to limit disulfide bonding and alkylated using 55mM iodoacetamide. In-gel digestion was conducted using sequencing grade-modified trypsin in 50 mM ammonium bicarbonate at 37 °C overnight. The peptides were extracted twice using 1%

trifluoroacetic acid in a 50% acetonitrile aqueous solution for 30 minutes. The peptide extracts were centrifuged in a SpeedVac to reduce the volume and concentrate the samples.

For isotopic MS analysis, peptides were separated using a 60-minute gradient elution at a flow rate of 0.3mL/minute with a Thermo-Dionex Ultimate 3000 HPLC system, which was directly interfaced with a Thermo LTQ-Orbitrap Velos Pro mass spectrometer. The analytical column was a homemade fused silica capillary column (75 mm ID, 150 mm in length; Upchurch, Oak Harbor, WA) packed with C-18 resin (300A, 5 mm; Varian, Lexington, MA). Mobile phase A contained 0.1% formic acid, and mobile phase B contained 100% acetonitrile supplemented with 0.1% formic acid. An LTQ-Orbitrap mass spectrometer was operated using the data-dependent acquisition mode in Xcalibur-2.0.7 software. A single full-scan mass spectrum in the Orbitrap (400-1800 m/z, 30,000 resolution) was followed by 20 data-dependent MS/MS scans in an ion trap at 35% normalized collision energy (CID).

The raw data were examined using MaxQuant (version 1.6.2.3) with standard settings and additional options retained to match between runs (between triplicates) with LFQ and iBAQ selected. To determine the stoichiometry between E2p and E3BP, we compared the relative abundances of the identified interactors as measured via the iBAQ intensities. The sequences (E2p residues 135 to 143 and E3BP residues 149 to 157) were used to query corresponding samples to estimate the stoichiometry of core subunits. The data are outlined in Fig. 1F.

**Protein interaction assay**

To examine the assembly mode of PDHc components, peripheral subunits and binding domains of core subunits were prepared individually and applied to a GST-pull down assay. For E1p and E3 isolation, intact PDH complexes were disassembled by incubating with 1.5 M sodium chloride on ice for 1 hour. Peripheral subunits were separated from core subunits via gel filtration chromatography (Superose 6, 10/300 GL, GE). Fractions containing E1p/E3 were identified by SDS-PAGE and desalinized into a physiological condition mimicking the mitochondrial matrix (20 mM Tris, pH 8.0; 150 mM sodium chloride) through repeated dialysis.

For expression of E2p and E3BP domains, truncated genes were *de novo* synthesized, including: E2p-LD1 (residues 91-167), E2p-LD2 (residues 218-294), E2p di-domain (residues 87-393), E2p-PSBD (residues 356-393), E3BP-LD (residues 54-130), and E3BP-PSBD (residues 180-217). These truncated genes were then cloned into the pGEX-6P-1 vector, which contains an N-terminal GST tag (GE Healthcare Life Sciences). The recombinant plasmids were transformed into *E. coli* strain BL21 (DE3). Bacteria cells were grown at 37 °C to mid-log phase, and expression was induced through the addition of 0.5 mM IPTG at 16 °C for 18 hours. Harvested cells were resuspended in 1×PBS

and disrupted by mechanical disruption and the cellular debris was removed completely through centrifugation at 13,000 rpm for 50 minutes. Fusion proteins in the lysate were purified using GST-affinity chromatography, as previously described. Approximately 100 μg of each type of E2p/E3BP domain was immobilized within 50 μL of glutathione agarose beads and equilibrated prior to being incubated together with 50 μg of E1p/E3 proteins at 4 °C for 90 minutes under gentle rotation. After thoroughly washing with 20 mL PBS, the bound proteins were eluted using elution buffer (10 mM reduced glutathione in PBS, pH 8.0) and analyzed via immunoblotting.

Equal amounts of protein were separated using SDS-polyacrylamide gel electrophoresis at 120 V for 1.5 hours. We then transferred the protein from the gels to PVDF membranes (Millipore, IPVH00010). The membranes were incubated with appropriate dilutions of primary antibodies overnight at 4 °C after blocking with 5% skim milk powder (Oxoid, UK) in 50 mL of TBST. The membranes were incubated with secondary antibodies conjugated to HRP for 1 hour at room temperature, followed by treatment with Clarity Max™ Western ECL Substrate (Bio-Rad, USA).

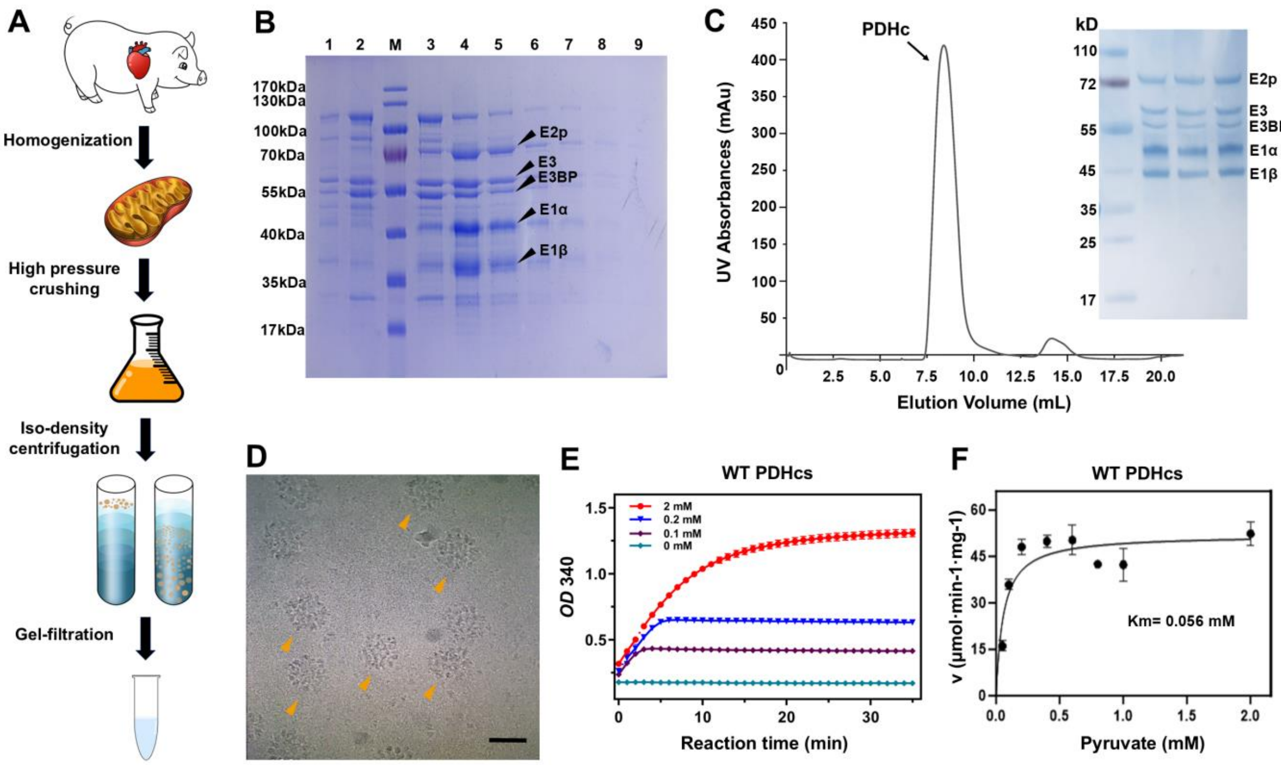


**Fig. S1 Protein purification of endogenous *Sus scrofa* PDHc**

A. Purification process schematic of endogenous PDHc from porcine heart mitochondria.

B. SDS-PAGE results of mitochondrial OADHcs isolation after sucrose density gradient centrifugation. Sample collection size per tube was 1 mL. Numbers above each lane stand for the tube number of collected samples. Lane 4 to 7 contains protein bands of each PDHc components which were individually validated by mass spectrum.

C. Size-exclusion chromatography (SEC) of purified porcine PDH complex. The peak corresponding to PDHc was subjected to SDS-PAGE separation. The protein band corresponding to each PDHc component is indicated, and standard molecular weights are labeled.

D. Representative cryo-EM micrograph of purified PDHc. Individual particles are marked by orange arrows. The black bar represents 50 nm.

E. Spectrophotometry analysis aiming at the total oxidative decarboxylation of pyruvate.

F. Michaelis-Menten plots for overall reaction of purified PDHc. The determined *K*m value (0.056 mM) is comparable with that of *Bos taurus* PDHc (0.072 mM) (Liu and Bisswanger, 2005)

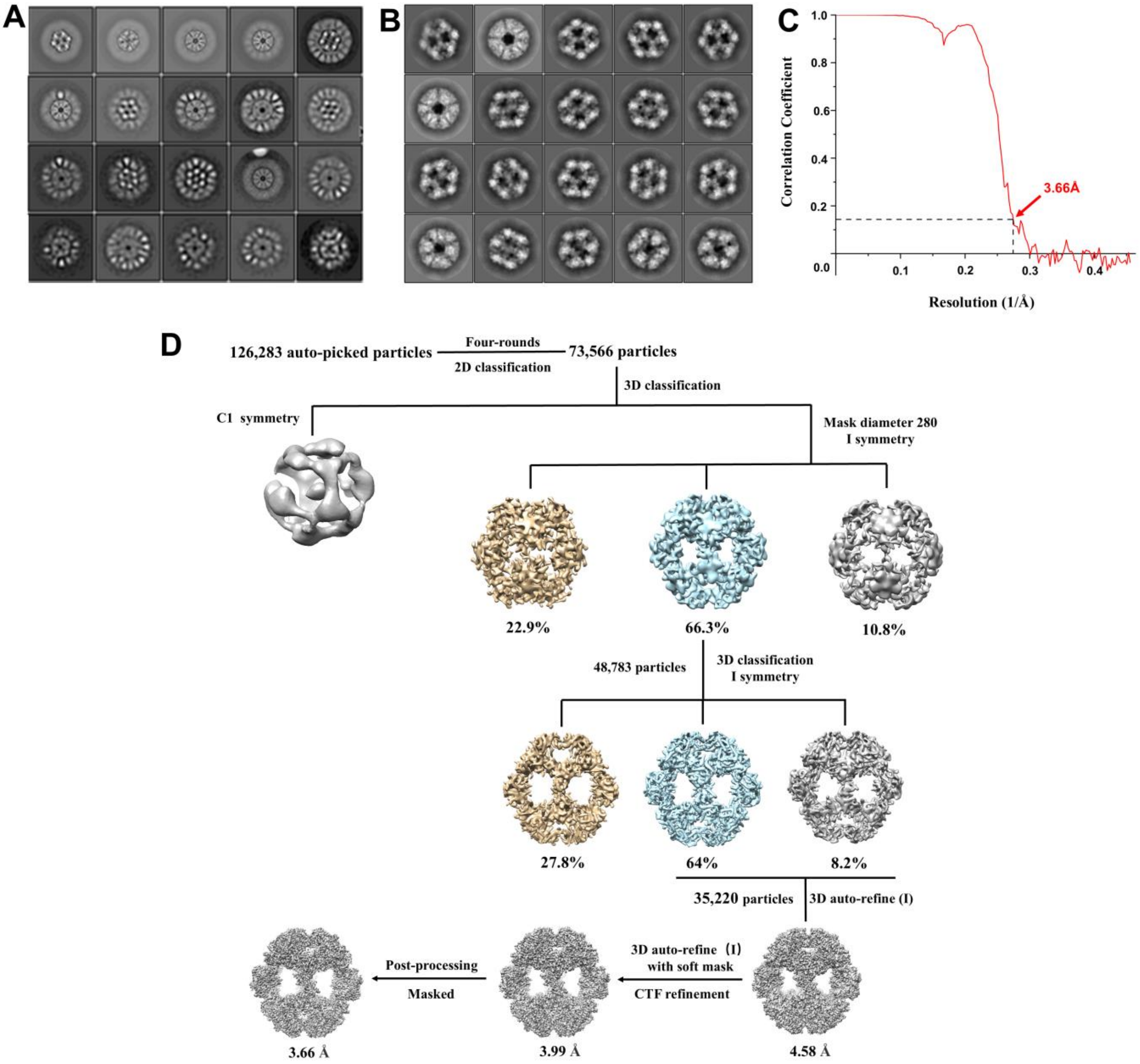


**Fig. S2 Cryo-EM image processing of PDHc**

A. Representative 2D class averages of intact PDHc.

B. Representative 2D class averages of PDHc inner core.

C. Resolution assessment for Cryo-EM core structure using Gold Standard Fourier Shell Correlation (FSC) plot from two 3D maps.

D. The workflow for 2D/3D reconstruction with PDHc cryo-EM data. In brief, a 400-Å mask was added to eliminate interference of exterior densities and 73k particles were selected after 2D classification, and subjected to two-round 3D classification with icosahedral symmetry. A final data set containing 35k particles were used for auto refinement and post-processing.

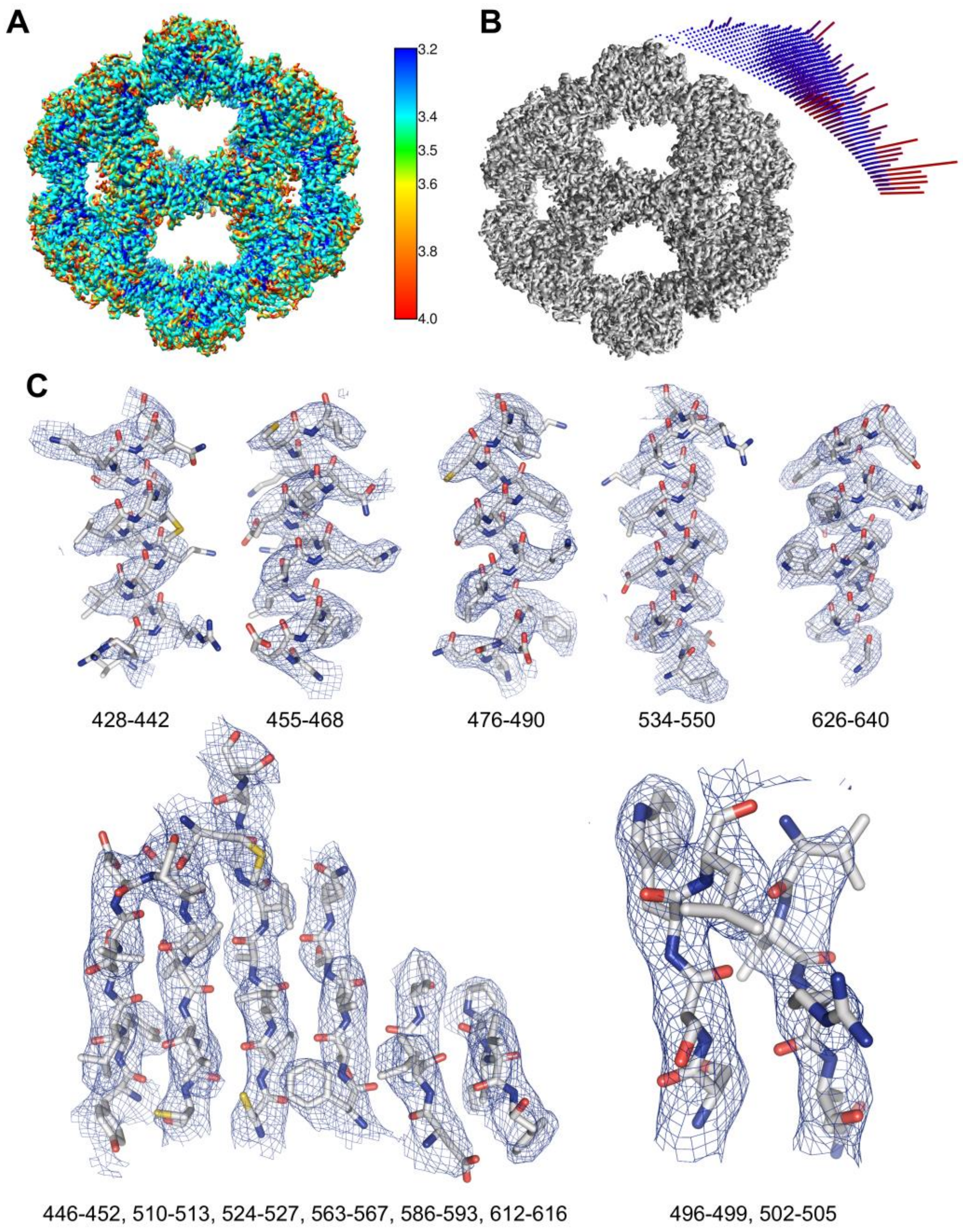


**Fig. S3 Structure determination and representative density map of PDHc core**

A. Local resolution estimation of PDHc inner core after the final density map. The map surface is colored according to local resolution.

B. Angular distribution of E2p-IC in core structure (symmetric element) in the final 3D auto-refinement reconstruction.

C. Density maps of representative regions. Stick-style atomic models (light grey) are fitted into the selected densities (blue mesh) and shown with the residue numbers of starting and ending amino acids. The density

maps were contoured at 5.5 σ.

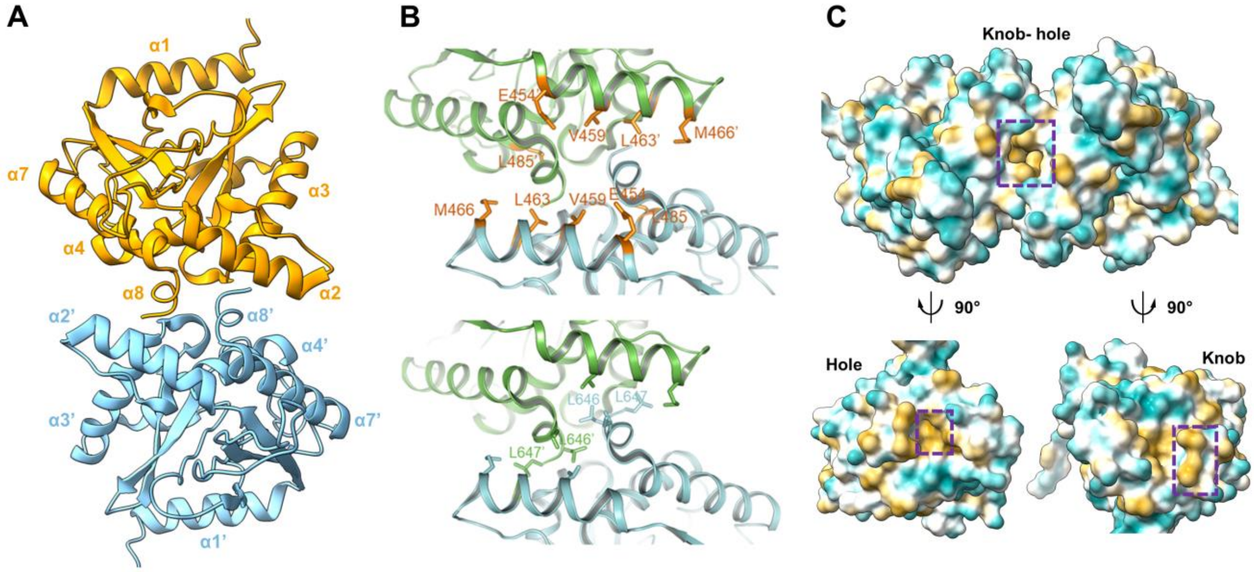


**Fig. S4 Molecular details of trimer-trimer interactions in dodecahedral E2p core**

A. Two-fold related dimer unit between scaffold vertex. The major secondary structures are labeled in each E2p-IC monomer, including α2, α4, α7, and α8, which are involved in trimer-trimer interactions.

B. Close-up view of trimer-trimer interface. Resides involved in hydrophobic environment formation are colored in orange and L646–L646' at the center of the 2-fold axis are colored in cyan and green.

C. Hydrophobic interactions between E2p trimers. The map surface of two E2p-IC in adjacent trimers are colored by their hydrophobicity index to show the double-handed “knob-hole” interaction.

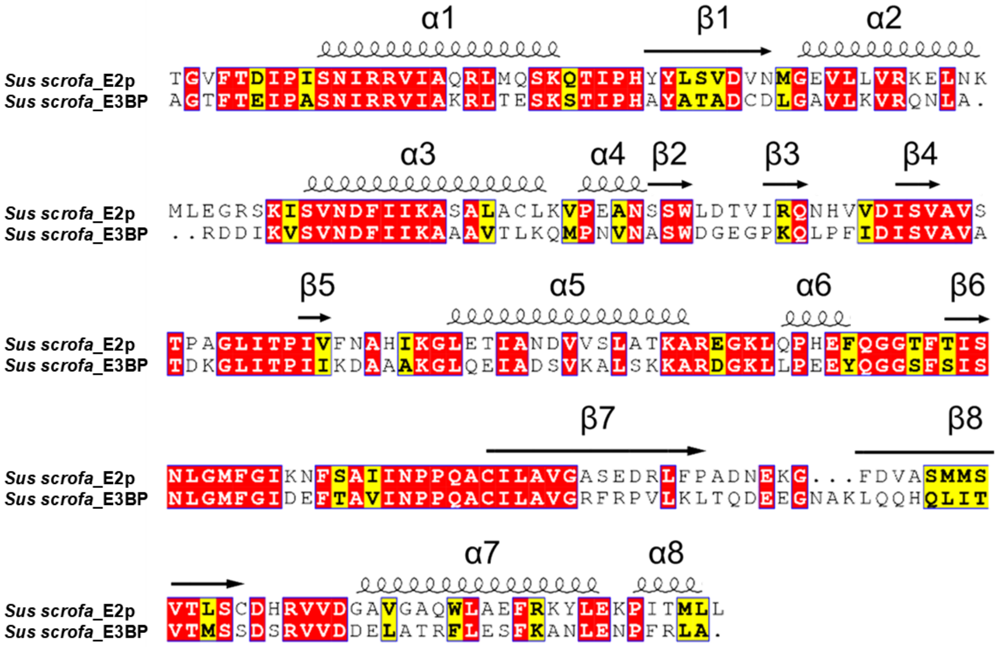


**Fig. S5 Sequence conservation of IC domains between porcine E2p and E3BP**

Sequence alignment of the last 230 amino acids between *Sus scrofa* E2p (UniProt ID: A0A4X1TBD2) and E3BP (UniProt ID: F1SGT3) using Clustal Omega and ESPript3. Conserved residues are colored in red and corresponding secondary structures are indicated.

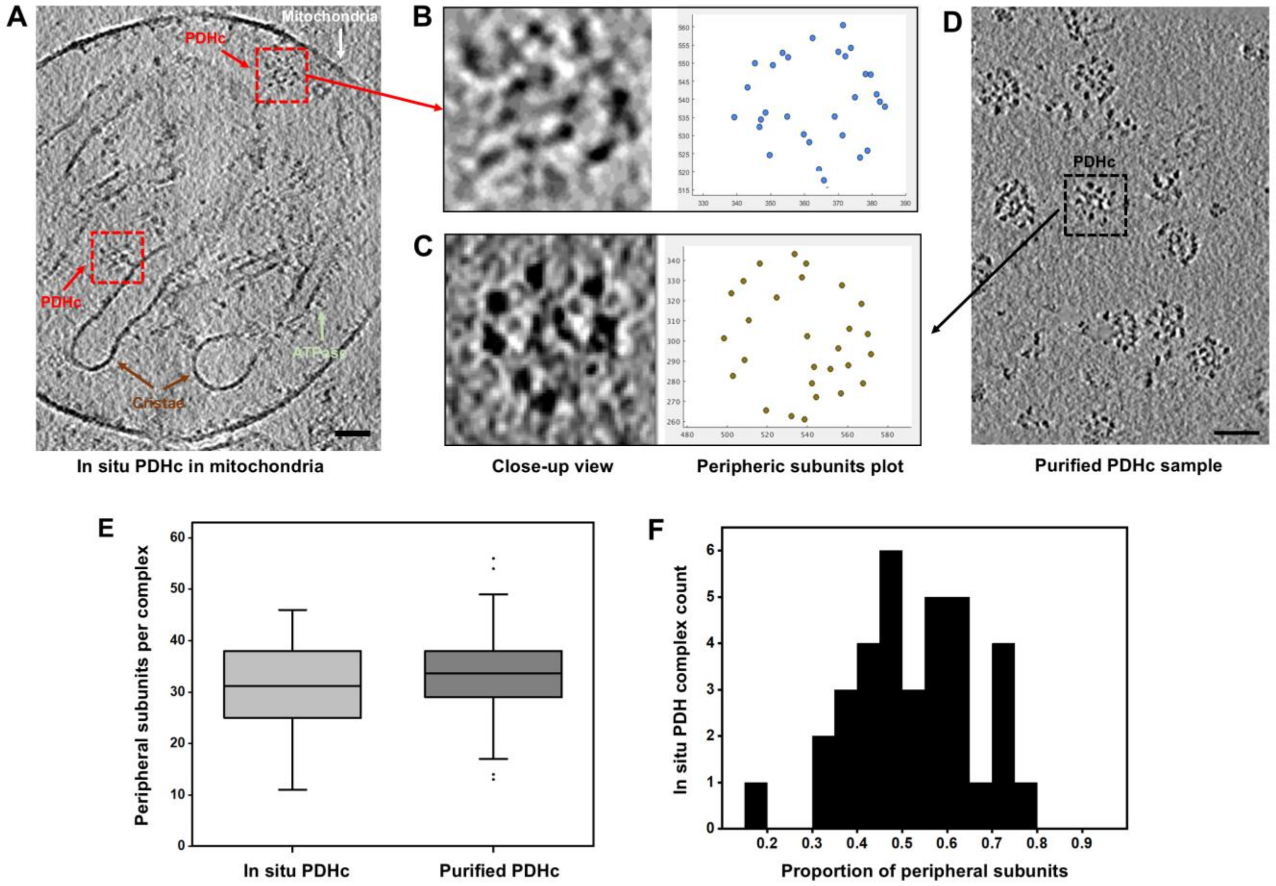


**Fig. S6 Comparison between in situ and purified PDHcs.**

A. A representative tomogram slice (5 Å thickness) of porcine mitochondria which is extracted from myocardial cell. The characteristic mitochondrial cristae (brown) and ATPase (light green) are marked. One of the in situ PDHc is indicated by red dashed box. The black bar represents 50 nm.

B. Left: Close-up view of the representative in situ PDHc. Right: Plot points of peripheral subunits in this complex. Each blue point represents a peripheric subunit.

C. Left: Close-up view of the purified PDHc. Right: Plot points of peripheral subunits in this complex. Each brown point represents a peripheric subunit.

D. A representative tomogram slice (5 Å thickness) of purified porcine PDHc which is consist with the tomogram shown in Fig 2A. One of PDHc is indicated by black dashed box. The black bar represents 50 nm.

E. Number of peripheral subunits per complex identified in in-situ and purified PDHc. Each in-situ complex contains 31±10 peripheral subunits.

F. Occupancy of core subunits in in-situ PDHc. The majority of in-situ cores is decorated by 40%-80% peripheral subunits, with an average value of 51%, which is comparable to that of purified samples (57%).

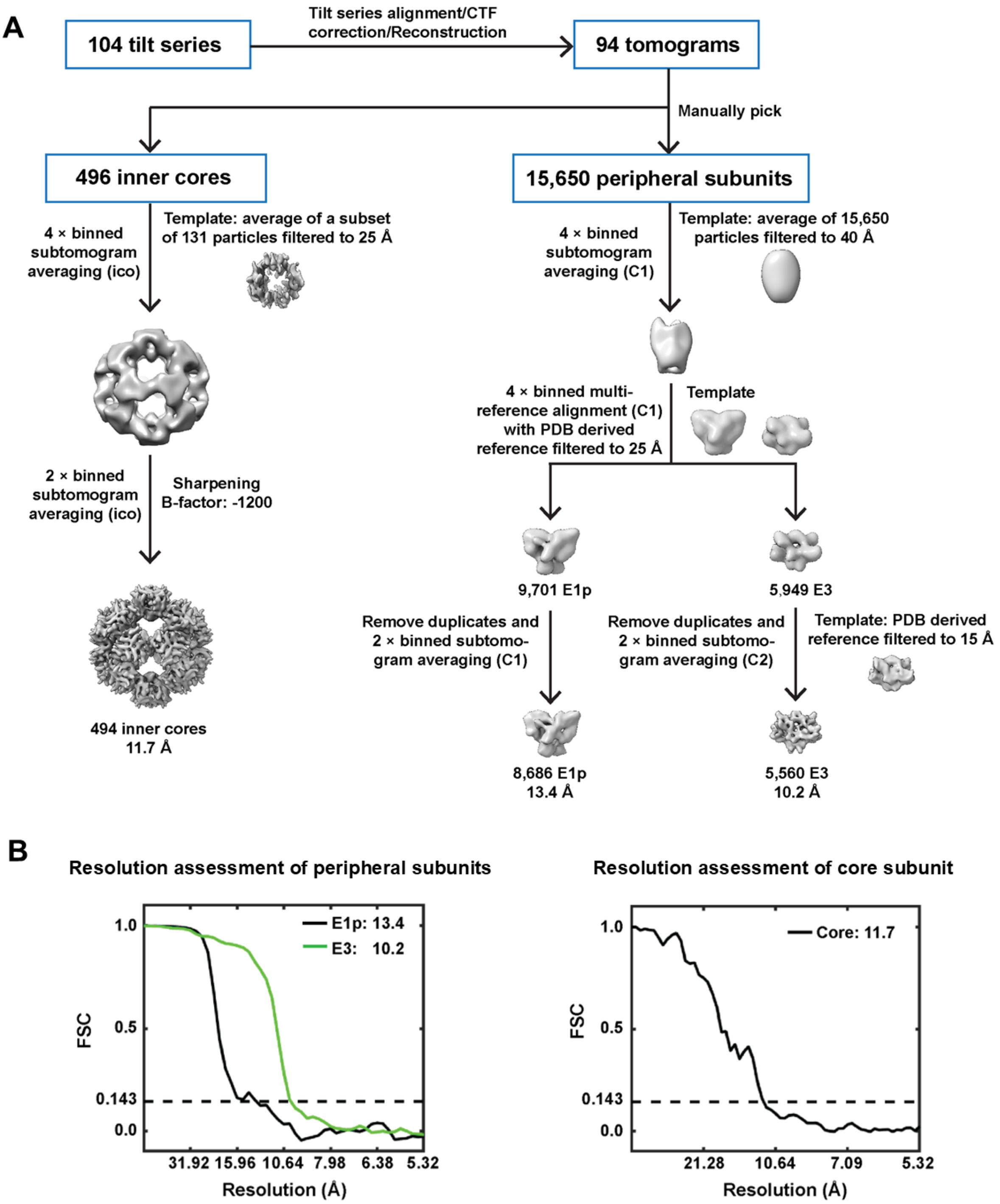


**Fig. S7 Cryo-ET data processing of purified *Sus scrofa* PDHc**

A. A workflow chart for STA reconstructions of individual PDHc components. Core subunits at the complex center and peripheral subunits around the core were separately processed. Subtomograms of peripheral subunits were classified into E1p and E3 using multi-reference classification before final reconstruction. For detailed description, please refer to the Methods.

B. Structural evaluation of STA reconstructions by Gold Standard Fourier Shell Correlation.

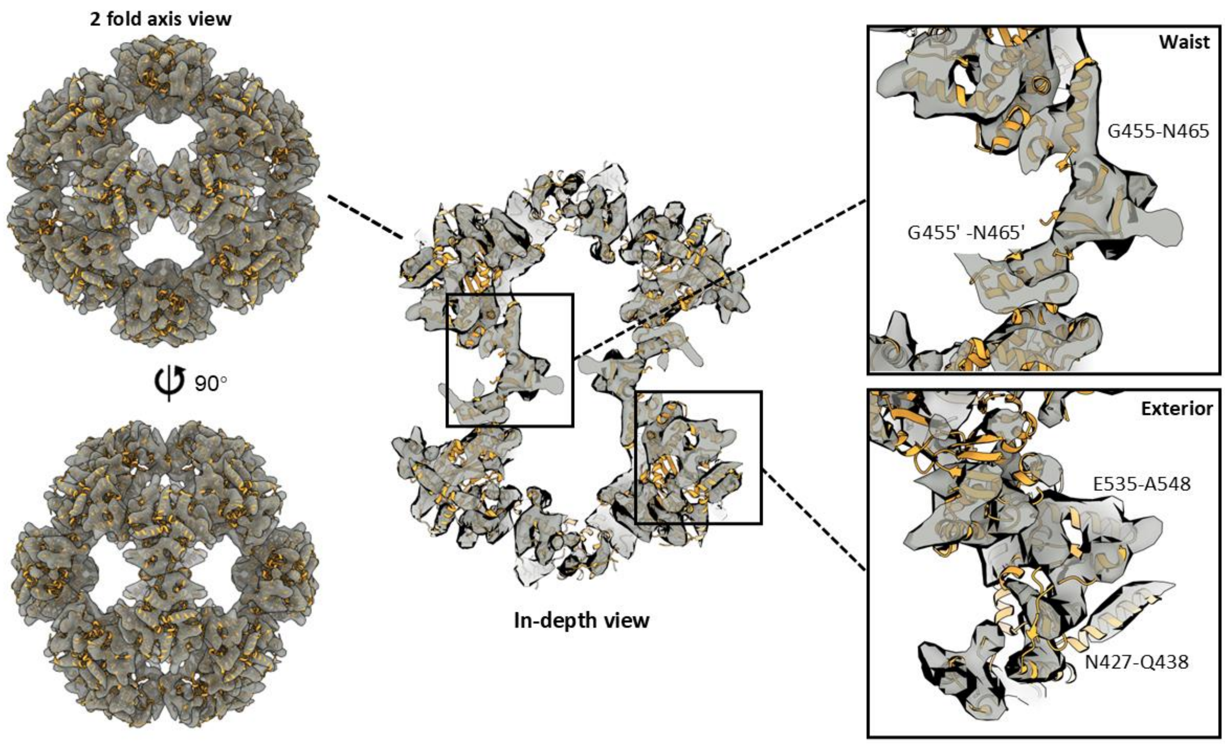


**Fig. S8 Cryo-ET structure of *Sus scrofa* E2p scaffold**

Left: Subtomogram averaging map (dark grey) fitted with E2p core model (orange, PDB ID: 7UOM) from a 2-fold axis view.

Middle: In-depth view of the STA reconstruction of core scaffold to show the exterior.

Right: Magnified view of the waist and exterior region marked in Middle. Close observation of ternary vertex surface (top) and flank (bottom).

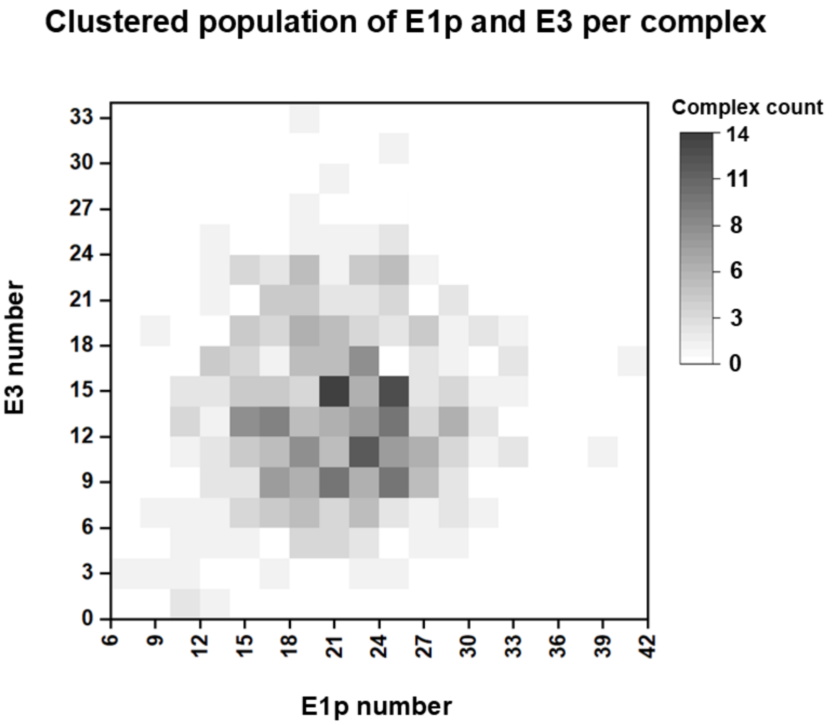


**Fig. S9 Quantitative distribution of peripheral subunits in one complex**

A 2D heat map reveals the clustered population distribution of peripheral subunit numbers on one single complex. The darker shaded blocks correspond to the population with more complexes. The most frequent population is (21 E1p + 15 E3), which is found in a total of 14 complexes.

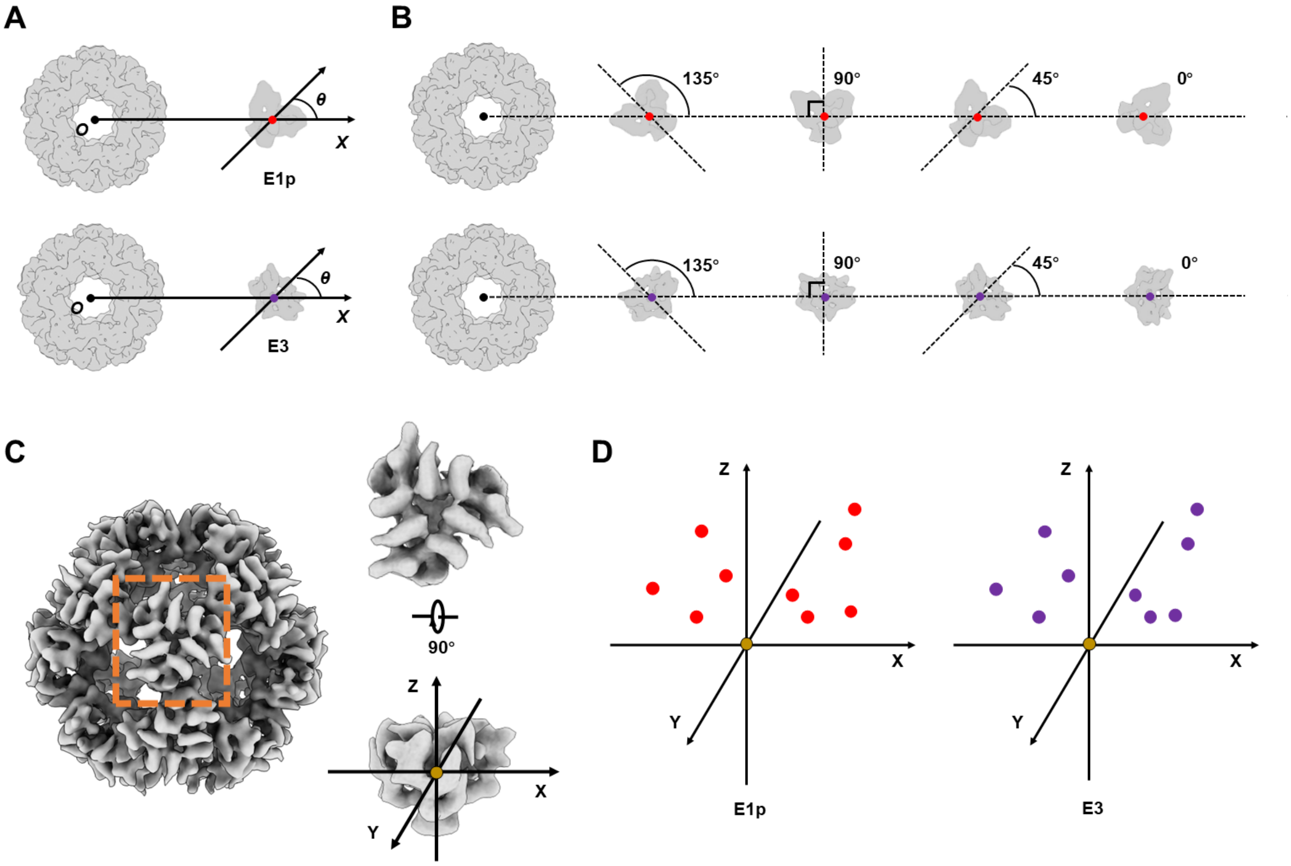


**Fig. S10 Spatial distribution analysis of peripheral subunits**

To define the quasi-polar coordinate system, a schematic diagram is presented in (A), wherein the center-of-mass of core scaffold is set as the origin and displayed as black point (*O*). Vectors pointing from the origin to the centers of E1p (red point) and E3 (purple point) are defined as reference axis ($O_X$). Angles between the reference axis and the twofold symmetry axis ($\theta$) of E1p and E3 represent their relative rotation to inner core. In particular, different orientations (0°, 45°, 90° and 135°) of E1p and E3 are shown as examples in (B). To analyze the distribution of E1p and E3 in local area, a three-dimensional coordinate system is established as in (C). The vertex of core scaffold, marked by orange dashed box, is magnified on the right. The center-of-mass of vertex is set as origin and the threefold axis of vertex is defined as Z axis. Two demonstrative coordinate systems are presented in (D), wherein the centers of E1p and E3 are displayed as red and purple points, respectively.

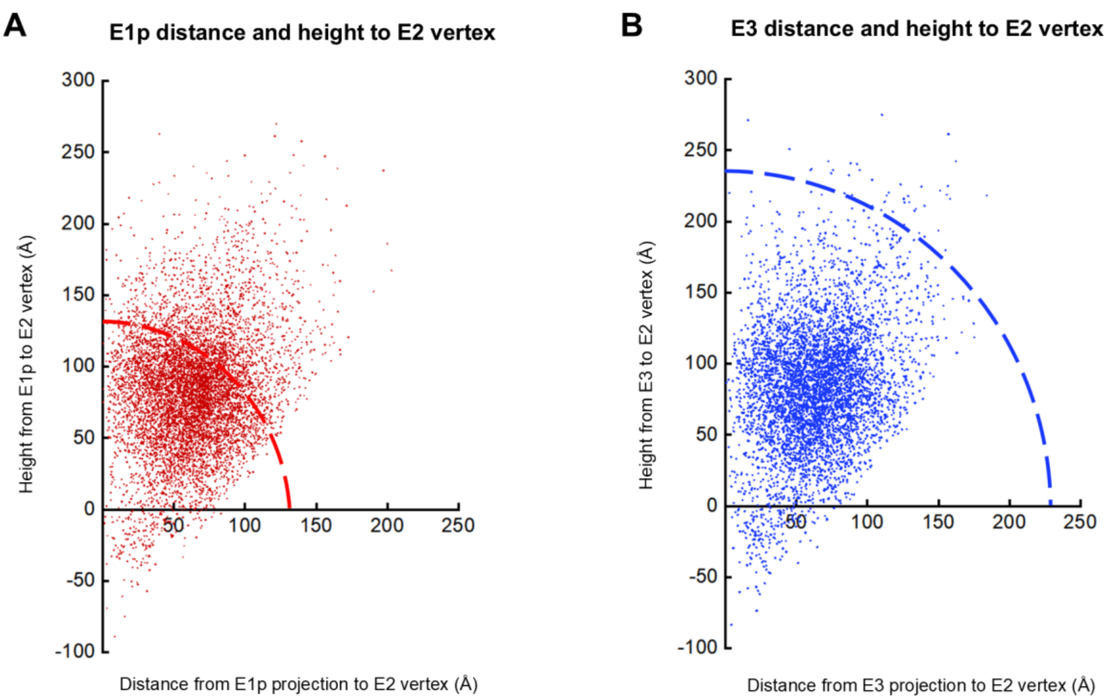


**Fig. S11 Distance distribution of peripheral subunits to neighboring E2p vertex**

The points in both plots represent individual projection of the mass of E1p (red) and E3 (blue). The expected tether lengths of a fully extended linker polypeptide are shown as red (E1p) and blue (E3) dashed lines in (A) and (B), respectively. Points to the outward of dashed lines are considered as the exceeding limit projections.

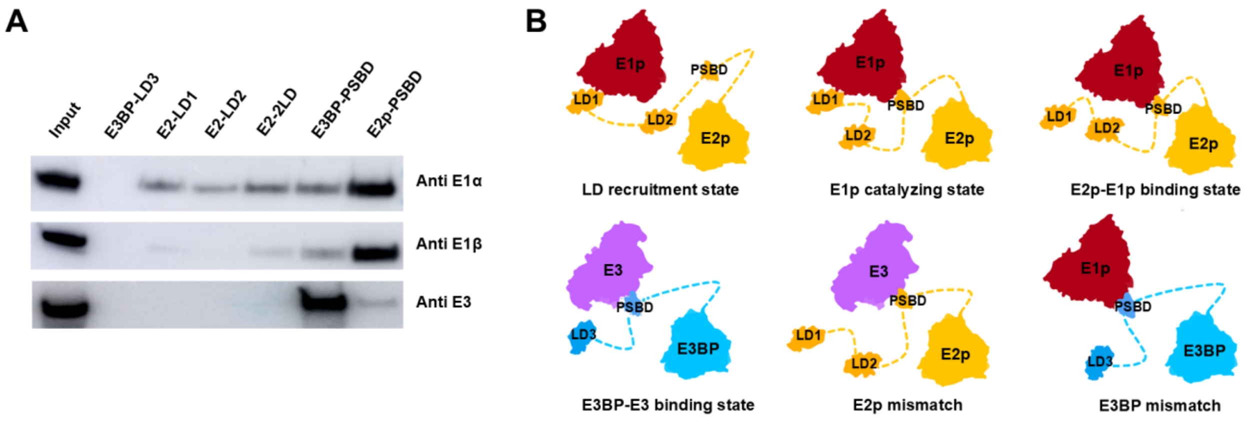


**Fig. S12 Proposed assembly mechanism of mammalian PDHc**

A. In vitro pull-down assay indicating additional interactions among PDHc components. Top: Schematic representation of Sus scrofa E2p and E3BP domains. Bottom: Western blot analysis of protein interactions between E1p/E3 and E2p/E3BP domains. Equal quantities of E2p/E3BP N-terminal domains tagged with GST were expressed in vitro and incubated with endogenous E1p/E3, which were disassembled from native complexes as the experimental subjects. The binding affinity of E3BP-LD (lane 2), E2P-LD1 (lane 3), E2p-LD2 (lane 4), E2p-LD1-LD2 (lane 5), and PSBDs (lane 6-7) to E1p/E3 was detected and visualized using corresponding subunit-specific antibodies. Lane1 represents an equal amount of E1p/E3 as the input in tested groups.

B. Proposed patterns of combining inner and outer components as deduced from the conclusions.

**Table S1. Cryo-EM data collection, refinement and validation statistics**

| | E2p |
|---|---|
| **Data collection and processing** | |
| Magnification | 130,000 |
| Voltage (kV) | 300 |
| Electron exposure ($e^-/Å^2$) | 48 |
| Defocus range (μm) | -1.5 ~ -2.5 |
| Pixel size (Å) | 1.0975 |
| Software | RELION 3.0 |
| Symmetry imposed | I1 |
| Initial particle images (no.) | 126,283 |
| Final particles images (no.) | 35,220 |
| Map resolution (Å) | 3.658 |
| FSC threshold | 0.143 |
| Local map resolution range (Å) | 4.0-3.2 |
| **Refinement** | |
| Software | PHENIX 1.14 |
| Model resolution (Å) | 3.6/3.7 |
| FSC threshold | 0.143/0.5 |
| Map sharpening *B* factor | -233 |
| Model composition | |
| Non-hydrogen atoms | 106,440 |
| Protein residues | 13,860 |
| Ligand | 0 |
| B factors ($Å^2$) | |
| Protein | 65.38 |
| Ligand | 0 |
| R.m.s deviations | |
| Bond length (Å) | 0.009 |
| Bond angles (°) | 1.328 |
| Validation | |
| MolProbity score | 1.82 |
| Clashscore | 1.24 |
| Poor rotamers (%) | 1.52 |
| Ramachandran plot | |
| Favored (%) | 96.92 |
| Allowed (%) | 3.08 |
| Disallowed (%) | 0 |

**Table S2. Cryo-ET data collection, refinement and validation statistics**

| **Data collection and processing** | | | |
|---|---|---|---|
| Voltage (kV) | 300 | | |
| Detector | Gantan K2 | | |
| Defocus range (µm) | -2.0 ~ -4.0 | | |
| Pixel size (Å) | 0.665 (super-resolution) | | |
| Tilt scheme | Bidirectional scheme | | |
| Number of tilt series | 104 | | |
| Tilt angle | -60° to +60° | | |
| Angle increment | 3° | | |
| Software | SerialEM | | |
| **Reconstruction** | E2p | E1p | E3 |
| Software | Dynamo 1.1.333 | | |
| Number of particles | 494 | 8,686 | 5,560 |
| **Symmetry imposed** | I | C1 | C2 |
| Model resolution (Å) | 11.7 | 13.4 | 10.2 |
| FSC threshold | 0.143 | 0.143 | 0.143 |
| Reconstruction pixel size (Å) | 2.66 | 2.66 | 2.66 |
| Map sharpening *B* factor | -1200 | N/A | N/A |

# Reference

Adams, P.D., Afonine, P.V., Bunkóczi, G., Chen, V.B., Davis, I.W., Echols, N., Headd, J.J., Hung, L.-W., Kapral, G.J., Grosse-Kunstleve, R.W.*, et al.* (2010). PHENIX: a comprehensive Python-based system for macromolecular structure solution. Acta Crystallographica Section D Biological Crystallography 66, 213-221.

Brautigam, C.A., Wynn, R.M., Chuang, J.L., Machius, M., Tomchick, D.R., and Chuang, D.T. (2006). Structural insight into interactions between dihydrolipoamide dehydrogenase (E3) and E3 binding protein of human pyruvate dehydrogenase complex. Structure 14, 611-621.

Castaño-Díez, D., Kudryashev, M., Arheit, M., and Stahlberg, H. (2012). Dynamo: A flexible, user-friendly development tool for subtomogram averaging of cryo-EM data in high-performance computing environments. Journal of Structural Biology 178, 139-151.

Emsley, P., and Cowtan, K. (2004). Coot: model-building tools for molecular graphics. Acta Crystallographica Section D Biological Crystallography 60, 2126-2132.

Goddard, T.D., Huang, C.C., Meng, E.C., Pettersen, E.F., Couch, G.S., Morris, J.H., and Ferrin, T.E. (2018). UCSF ChimeraX: Meeting modern challenges in visualization and analysis. Protein Science 27, 14-25.

Grant, T., and Grigorieff, N. (2015). Measuring the optimal exposure for single particle cryo-EM using a 2.6 Å reconstruction of rotavirus VP6. Elife 4, e06980.

Kato, M., Wynn, R.M., Chuang, J.L., Tso, S.C., Machius, M., Li, J., and Chuang, D.T. (2008). Structural basis for inactivation of the human pyruvate dehydrogenase complex by phosphorylation: role of disordered phosphorylation loops. Structure 16, 1849-1859.

Lengyel, J.S., Stott, K.M., Wu, X., Brooks, B.R., Balbo, A., Schuck, P., Perham, R.N., Subramaniam, S., and Milne, J.L. (2008). Extended polypeptide linkers establish the spatial architecture of a pyruvate dehydrogenase multienzyme complex. Structure 16, 93-103.

Liu, X., and Bisswanger, H. (2005). Interaction of thiamin diphosphate with phosphorylated and dephosphorylated mammalian pyruvate dehydrogenase complex. Biol Chem 386, 11-18.

Liu, Y.T., Zhang, H., Wang, H., Tao, C.L., Bi, G.Q., and Zhou, Z.H. (2022). Isotropic reconstruction for electron tomography with deep learning. Nat Commun 13, 6482.

Mastronarde, D.N. (2005). Automated electron microscope tomography using robust prediction of specimen movements. Journal of Structural Biology 152, 36-51.

Pettersen, E.F., Goddard, T.D., Huang, C.C., Couch, G.S., Greenblatt, D.M., Meng, E.C., and Ferrin, T.E. (2004). UCSF Chimera?A visualization system for exploratory research and analysis. Journal of Computational Chemistry 25, 1605-1612.

Tegunov, D., and Cramer, P. (2019). Real-time cryo-electron microscopy data preprocessing with Warp. Nature Methods 16, 1146-1152.

Turoňová, B., Schur, F.K.M., Wan, W., and Briggs, J.A.G. (2017). Efficient 3D-CTF correction for cryo-electron tomography using NovaCTF improves subtomogram averaging resolution to 3.4 Å. Journal of Structural Biology 199, 187-195.

Xu, K., Wang, Z., Shi, J., Li, H., and Zhang, Q.C. (2019). A^2-Net: Molecular Structure Estimation from Cryo-EM Density Volumes. ArXiv abs/1901.00785.

Yao, H., Song, Y., Chen, Y., Wu, N., Xu, J., Sun, C., Zhang, J., Weng, T., Zhang, Z., Wu, Z.*, et al.* (2020). Molecular Architecture of the SARS-CoV-2 Virus. Cell 183, 730-738.e713.

Zhang, K. (2016). Gctf: Real-time CTF determination and correction. Journal of Structural Biology 193, 1-12.

Zheng, S., Wolff, G., Greenan, G., Chen, Z., Faas, F.G.A., Bárcena, M., Koster, A.J., Cheng, Y., and Agard, D.A. (2022). AreTomo: An integrated software package for automated marker-free, motion-corrected cryo-electron tomographic alignment and reconstruction. J Struct Biol X 6, 100068.

Zheng, S.Q., Palovcak, E., Armache, J.-P., Verba, K.A., Cheng, Y., and Agard, D.A. (2017). MotionCor2: anisotropic correction of beam-induced motion for improved cryo-electron microscopy. Nature Methods 14, 331-332.

Zivanov, J., Nakane, T., Forsberg, B.O., Kimanius, D., Hagen, W.J.H., Lindahl, E., and Scheres, S.H.W. (2018). New tools for automated high-resolution cryo-EM structure determination in RELION-3. eLife 7.
